# Risk Factors and Outcome Analysis of Gram-Positive Bacteremia in Critically Ill Patients

**DOI:** 10.7759/cureus.36585

**Published:** 2023-03-23

**Authors:** Navpreet Singh, Sandeep Puri, Anshul ., Sachin Kumar, Hardik Pahuja, Rajni Kalia, Rashmi Arora

**Affiliations:** 1 Internal Medicine, Gian Sagar Medical College and Hospital, Rajpura, IND; 2 Internal Medicine, Dayanand Medical College & Hospital, Ludhiana, IND; 3 Anesthesiology, Pandit Bhagwat Dayal Sharma Post Graduate Institute of Medical Sciences, Rohtak, IND; 4 Anaesthesiology, All India Institute of Medical Sciences, New Delhi, Delhi, IND; 5 Psychiatry, Gian Sagar Medical College and Hospital, Rajpura, IND; 6 Anaesthesiology, Government Medical College & Hospital, Chandigarh, IND; 7 Anaesthesiology, Pandit Bhagwat Dayal Sharma Post Graduate Institute of Medical Sciences, Rohtak, IND

**Keywords:** mrsa, staphylococcus aureus, gram positive bacteremia, icu, bloodstream infections

## Abstract

Introduction

Bloodstream infection (BSI) is a common problem for patients in the intensive care unit (ICU). Nearly 60% of primary bloodstream infections are caused by Gram-positive cocci. Gram-positive bacteria gain access to the bloodstream through invasive procedures and various patient care equipment like catheters, intravenous lines, and mechanical ventilators. S. aureus is considered to be the major cause of septicemia. Knowledge of healthcare-associated infections and the antimicrobial susceptibility patterns of the isolates are crucial in guiding empirical treatment.

Methods

This prospective observational study was conducted in Medical ICU, Dayanand Medical College & Hospital, Ludhiana over a period of one year (December 2015 to November 2016). Patients whose blood cultures tested positive for Gram-positive bacteria were included in the study. This study was carried out to assess the implications and risk factors for nosocomial BSI and several factors, including the age of the patient, the severity of illness, the presence of catheters, and the microorganisms causing the BSI to independently predict mortality. Chief complaints and risk factors were evaluated. APACHE-II scores were calculated for all patients and outcomes were analyzed.

Results

In our study, the mean age of patients was 50.93±14.09 years. Central line insertion was found as the most common risk factor (58.7%). A statistically significant correlation was obtained between APACHE-II scores and the presence of risk factors i.e. central line insertion (p-value=0.010) and diabetes mellitus (p-value=0.003). The most common Gram-positive pathogen isolated by blood culture was methicillin-sensitive S. aureus (44.2%). For management, the majority of the patients were prescribed teicoplanin (58.7%). The 28-day overall mortality rate in our study was 52.9%.

Conclusion

We conclude that independent risk factors like diabetes mellitus, central line insertion, and acute pancreatitis in adult patients with Gram-positive bacteremia were associated with higher mortality. We have also concluded that the administration of early appropriate antibiotics improves patient outcomes.

## Introduction

Bloodstream infection (BSI) is a leading complication in ICU patients [1]. The epidemiology of BSIs is changing with the evolution of medical care. BSI can be caused by either Gram-positive bacteria or Gram-negative bacteria [2]. Nearly 60% of primary BSIs are caused by Gram-positive cocci; of these, S. aureus is a major cause of septicemia [3-4].

BSI results from patients' endogenous flora, which gains access to the bloodstream through various patient care equipment like catheters, intravenous lines, and mechanical ventilators [5-6]. Failure to follow proper sterilization and disinfectant guidelines increases the prevalence of BSI [7].

Gram-positive sepsis is an important infectious disease problem in hospitals, especially in critical care patients. The emergence of resistance to multiple antimicrobial agents in pathogenic bacteria has been declared a public health threat by World Health Organization (WHO) [8]. Irrational use of antibiotics is a significant factor for resistance [9]. Mostly the selection of antibiotics depends upon the site of infection, resistance pattern in that particular region, and probable causative organism [10-11]. The type of bacteria causing infections and their susceptibility patterns have been found to vary from one setting to another, a fact which highlights the importance of having local surveillance data for planning and implementing infection prevention and control measures [12-14].

The epidemiology of healthcare-associated infections (HCAIs) and the antimicrobial susceptibility pattern of the isolates are crucial for guiding empirical treatment [15]. Hence, an observational study was conducted to know the risk factors and clinical outcomes of Gram-positive bacteremia in an ICU setting and to compare drug-sensitive and resistant strains.

## Materials and methods

This is a prospective cohort observational study, conducted in the ICU of the Department of Medicine, Dayanand Medical College & Hospital, Ludhiana from December 2015 to November 2016, after receiving approval from the institutional ethical committee vide letter no. DMCH/4/18-2015 and informed written consent from patients/attendants. Adult patients (aged more than 18 years) whose blood culture tested positive for Gram-positive bacteria were included in the study. Patients whose blood culture was sterile or positive for Gram-negative bacteria were excluded.

A predefined proforma was used as a tool for data collection which included the demographic characteristics and medical history of the patient. Presenting complaints and diagnoses at the time of admission were recorded. Risk factors for BSI like any underlying disease, central venous line, tracheostomy, history of any prior antibiotic use in the last 90 days and arterial line were noted [16]. The antimicrobial sensitivity and resistance of the Gram-positive bacteria that were isolated while including the patient in the study were recorded. Patient outcome was recorded as discharged in stable condition, discharged against medical advice (DAMA), and 28-day mortality. APACHE-II score was also calculated.

Statistical analysis

Data was described in terms of range; mean ±standard deviation (± SD), frequencies (number of cases), and relative frequencies (percentages) as appropriate. To determine whether the data were normally distributed, a Kolmogorov-Smirnov test was used. For quantitative variables, a Student t-test and Mann-Whitney U test/Kruskal-Wallis test were used for parametric and non-parametric data respectively. For comparing categorical data, a chi-square (χ2) test was performed and the Fisher exact test was used when the expected frequency was less than 5.

Covariates obtaining a p-value <0.05 in the univariate analyses were included in the multivariate binary logistic regression analysis. A probability value (p-value) less than 0.05 was considered statistically significant. All statistical calculations were done using IBM SPSS for Windows v.21 (IBM Corp., Armonk, NY). 

## Results

A total of 104 patients were included in the study. Out of 104 subjects, 75 (72.1%) were males and 29 (27.9%) were females. Most of the patients (48%) belonged to the age group of 41-60 years with a mean age of 50.93 ± 14.08 years. The minimum age reported was 21 years while the maximum age was 85 years. The various presenting complaints recorded in our patients were fever, shortness of breath, pain abdomen, altered sensorium, yellow discoloration of eyes, abdominal distension, and cough. Of these, fever was the most common presenting complaint (71.2%).

Figure 3 shows the distribution of patients as per the diagnosis.

**Figure 1:**
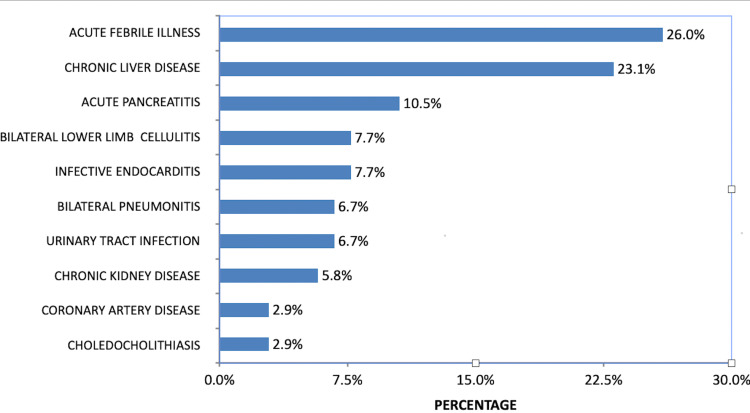
Distribution of patients as per the diagnosis

Around 35.6% of patients had a history of antecedent antibiotic use before admission. Central line insertion (58.7%) was the most common risk factor, followed by diabetes mellitus (47.1%).

**Table 1:** DISTRIBUTION OF RISK FACTORS IN THE PATIENTS DM: diabetes mellitus, HTN: hypertension, CLD: chronic liver disease, CAD: coronary artery disease, HCV: hepatitis C virus, COPD: chronic obstructive pulmonary disease, CKD: chronic kidney disease

Risk factors	No. of patients	% age
Central line	61	58.7%
Type 2 DM	49	47.1%
Renal failure	45	43.3%
Arterial line	36	34.6%
HTN	34	32.7%
CLD	24	23.1%
Tracheostomy	7	6.7%
HCV+	5	4.8%
CAD	5	4.8%
CKD	4	3.8%
COPD	4	3.8%

S. aureus (66.3%) was found to be the most offending pathogen (Figure 1).

**Figure 2:**
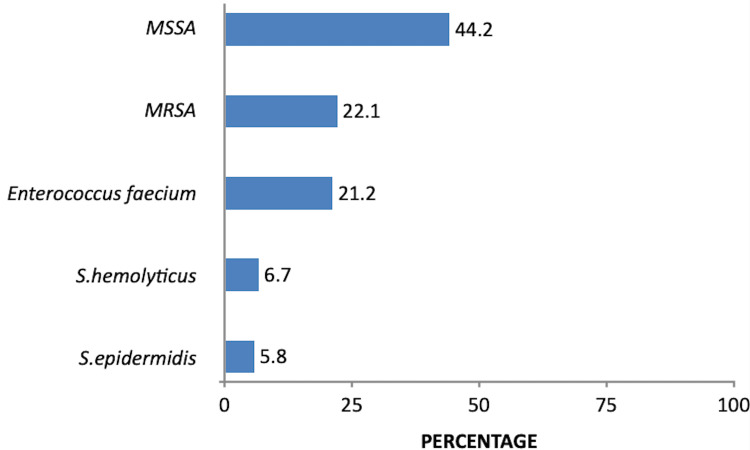
Gram-positive bacteria isolated from blood samples MSSA: methicillin-sensitive Staphylococcus aureus, MRSA: methicillin-resistant Staphylococcus aureus, S. hemolyticus: Staphylococcus hemolyticus, S. epidermidis: Staphylococcus epidermidis

Antimicrobial susceptibility pattern showed that all isolates of MSSA, MRSA, Enterococcus, S. hemolyticus, and S. epidermidis were sensitive to vancomycin, teicoplanin, and linezolid whereas resistance pattern was different in each bacteria

**Table 2:** Antimicrobial sensitivity of different Gram-positive bacteria MSSA: Methicillin-sensitive staphylococcus aureus, MRSA: methicillin-resistant staphylococcus aureus, S. hemolyticus: Staphylococcus hemolyticus, S. epidermidis: Staphylococcus epidermidis, PEN: penicillin, GEN: gentamicin, CIPRO: ciprofloxacin, LEVO: levofloxacin, ERY: erythromycin, CLINDA: clindamycin, LZ: linezolid, DAPTO: daptomycin, TEICO: teicoplanin, VAN: vancomycin, TETRA: tetracycline, RIF: rifampicin, COTRI: cotrimoxazole

Drugs	No. of isolates (% sensitivity)									
	MSSA (N=46)		MRSA (N=23)		Enterococcus faecium (N=22)		S. hemolyticus (N=7)		S. epidermidis (N=6)	
PEN	12	26.1%	1	4.3%	4	18.2%	2	28.6%	1	16.7%
GEN	17	37.0%	9	39.1%	2	9.1%	3	42.9%	1	16.7%
CIPRO	10	21.7%	1	4.3%	3	13.6%	0	0.0%	1	16.7%
LEVO	12	26.1%	1	4.3%	2	9.1%	3	42.9%	1	16.7%
ERY	16	34.8%	4	17.4%	6	27.3%	0	0.0%	0	0.0%
CLINDA	27	58.7%	12	52.2%	8	36.4%	3	42.9%	0	0.0%
LZ	46	100.0%	23	100.0%	22	100.0%	7	100.0%	6	100.0%
DAPTO	13	28.3%	18	78.3%	3	13.6%	5	71.4%	2	33.3%
TECIO	46	100.0%	23	100.0%	22	100.0%	7	100.0%	6	100.0%
VAN	46	100.0%	23	100.0%	18	81.8%	7	100.0%	6	100.0%
TETRA	19	41.3%	18	78.3%	5	22.7%	4	57.1%	2	33.3%
RIF	7	15.2%	18	78.3%	1	4.5%	4	57.1%	0	0.0%
COTRI	9	19.6%	5	21.7%	2	9.1%	2	28.6%	1	16.7%

Around 58.7% of patients received teicoplanin followed by vancomycin and linezolid. There was no statistically significant difference observed in outcome with regard to the type of antibiotic used (p-value 0.321).

**Figure 3:**
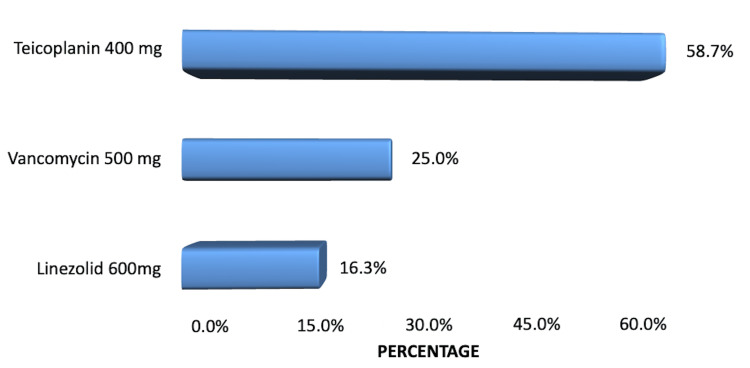
Antibiotics used for treatment

Around 89.4% of the patients had APACHE-II scores between 0-25 and 10.6% had APACHE-II scores of ≥26. The highest APACHE-II score was 33 and the minimum was 2 with a mean of 17.63 ± 7.07. A statistically significant correlation was obtained between the APACHE-II score and the presence of risk factors i.e. central line (p-value 0.010) and diabetes mellitus (p-value 0.003). No statistically significant correlation was obtained between APACHE SCORE-II and different kinds of diagnosis noted in our patients (p-value 0.264).

In our study, 35 (33.7%) cases showed improvement and were discharged in satisfactory condition, while 20 (19.2%) cases expired during their hospital stay. The remaining 49 patients were discharged against medical advice (DAMA); 35 (33.7%) out of these 49 DAMA patients expired during the 28-day follow-up period raising overall mortality to 52.9%.

There was no statistically significant difference in final outcome (28-day mortality versus discharge) in relation to demographic profile i.e.age (p-value 0.354) and gender (p-value 0.306). A statistically significant correlation was observed between risk factors i.e. central line insertion and diabetes mellitus and outcome indicating that 28 days mortality was more in these patients (p value<0.05). 74.5% of patients with a central line in situ and 61.8% of patients with type 2 diabetes mellitus expired during the study duration. A statistically significant correlation (p-value 0.015) was seen between patients diagnosed with acute pancreatitis and outcome indicating that 28-day mortality was higher among these patients. The type of organism isolated from blood culture was not related to final outcome (p value>0.05). The mean APACHE-II score in discharged patients was 14.08 ± 6.76 and in expired patients (28-day mortality) the score was 20.80 ± 5.76 (p value<0.05).

In our study, a statistically significant association was found between patients having central line insertion and mortality with a p-value of 0.022 and an exponential beta of 2.99, followed by APACHE SCORE-II with a p-value of zero and an exponential beta of 1.155 (Table 5). 

**Table 3:** Multivariate Analysis T2DM: type 2 diabetes mellitus, B: beta, SE: standard error, Wald: Wald test, df: degrees of freedom, Exp(B): exponential beta, CI: confidence interval

	B	SE	Wald	df	p-value	Exp(B)	95% C.I. for EXP(B)	
							Lower	Upper
Central line	1.095	0.480	5.208	1.000	0.022	2.990	1.167	7.658
T2 DM	0.738	0.484	2.323	1.000	0.127	2.091	0.810	5.398
APACHE SCORE II	0.144	0.040	13.269	1.000	0.000	1.155	1.069	1.248

## Discussion

BSI is a leading infectious complication among ICU patients, whose hospital stays are longer as compared to ward patients, resulting in excess morbidity and mortality [17]. Catheter-related bloodstream infections (CRBSI) increase mortality risk by 2.27 times [18].

Our demographic data showed that the mean age of patients was 51 years, which is consistent with studies by Pittet et al. and Vardakas et al. wherein the mean age was reported as 49 and 66 years, respectively [19-20].

The majority of patients had an acute febrile illness (26%) followed by chronic liver disease (23.1%) and acute pancreatitis (10.6%). These findings are similar to a study by Al-Hamzi et al. which reported that 22.9% of the BSIs were present in liver disease patients, 20.6% in urinary system disease patients, and 11.1% in prostate gland disease patients [21].

The most common Gram-positive pathogen isolated by blood culture is Methicillin-sensitive S. aureus (44.2%) followed by Methicillin-resistant S. aureus (22.1%), Enterococcus faecium (21.2%). Other organisms are Staphylococcus epidermidis and Staphylococcus hemolyticus. A comparable prospective study by Friedman et al. reported that 52% of BSI episodes in their study were caused by MRSA [22]. In 1992, MRSA accounted for 57% of all ICU-acquired S. aureus infections recorded in the European Prevalence of Infection in Intensive Care (EPIC) study [23].

The antimicrobial susceptibility profile of S. aureus showed that all of the isolates were sensitive to teicoplanin and vancomycin. These results are comparable to those of Cervera et al., Cağatay et al., and Erdem et al. [24-26]. Administration of early appropriate antibiotics is recognized as the most important intervention linked to improving patient outcomes in bacteremia and sepsis. Out of 104 patients in the present study, the majority were prescribed teicoplanin (58.7%) followed by vancomycin (25%) and linezolid (16.3%). There was no statistically significant difference observed in outcome and type of antibiotics (p-value 0.032).

Pitout and Laupland reported chances of infection are more with underlying diseases like renal failure, diabetes mellitus, and chronic liver disease, and these infections are mostly drug resistant. A study by Süner et al showed intubation, arterial catheter, tracheostomy, underlying diseases of chronic renal failure and diabetes mellitus, and high APACHE-II scores as major risk factors for bacteremia [4-27]. In our study, central line insertion (58.7%) is the most common risk factor, followed by diabetes mellitus (47.1%), renal failure (43.3%), an arterial line (34.6%), and hypertension (32.7%), while 6.7% cases had undergone tracheostomy.

For gauging the severity of illness, the APACHE-II score system was used in our study. It is observed that patients with higher APACHE-II scores have poor outcomes i.e. mortality (p-value <0.05). A study by Tao et al. showed that APACHE-II score >20 and the presence of >3 types of diseases were associated with earlier CRBSI onset [18]. Our study also showed a statistically significant association between central line insertion and mortality with a p-value of 0.022 and exponential beta of 2.99, followed by APACHE SCORE-II with a p-value of zero and exponential beta of 1.155.

Therefore, it is evident from the above discussion that BSI remains one of the most important causes of morbidity and mortality in ICU patients. S. aureus is a fearsome pathogen with significant morbidity and mortality. MRSA is common in the community and hospital, especially in the ICU. Patients who are elderly, immunosuppressed, and exposed to prolonged ICU care and antibiotics are at risk of colonization and subsequent infections. It is essential to have knowledge of the prevalence of BSI in respective ICUs as well as of its antibiotic sensitivity pattern so that empirical antibiotic choices can be made. Treatment customarily includes glycopeptide, vancomycin, or teicoplanin. Another alternative is linezolid.

Despite intensive infection control measures, the spread of BSIs in many ICUs is still an unresolved issue. Our study has tried to show the risk factors, sensitivities, and resistance patterns of Gram-positive infections so that effective measures can be implemented to reduce BSIs in ICU settings.

There are certain limitations in our study (a) the single-centre structure, (b) the relatively smaller sample size, and (c) the focus on Gram-positive bacteremia only. Future multicentric studies involving various cohorts with the aim of studying both Gram-positive as well as Gram-negative bacteremia are warranted.

## Conclusions

We conclude that independent risk factors like diabetes mellitus, central line insertion, and acute pancreatitis in adult patients with Gram-positive bacteremia were associated with higher mortality. Also, higher APACHE-II scores were associated with higher 28-day mortality. We hereby hypothesized that maintaining asepsis, normoglycemia, timely removal of the central catheter and appropriate antibiotic therapy are important measures for improving patient outcomes in bacteremia and sepsis.
